# Exacerbation of myopathy triggered by antiobesity drugs in a patient with multiple acyl-CoA dehydrogenase deficiency

**DOI:** 10.1186/s12883-021-02121-y

**Published:** 2021-02-27

**Authors:** Po-Yu Lin, Wen-Chen Liang, Wei-An Liao, Yuan-Ting Sun

**Affiliations:** 1 Department of Neurology, National Cheng Kung University Hospital, College of Medicine, National Cheng Kung University, Tainan, Taiwan; 2 Department of Pediatrics, Kaohsiung Medical University Hospital, Kaohsiung Medical University, Kaohsiung, Taiwan; 3 Department of Pediatrics, School of Medicine, College of Medicine, Kaohsiung Medical University, Kaohsiung, Taiwan; 4 Department of Pathology, National Cheng Kung University Hospital, College of Medicine, National Cheng Kung University, Tainan, Taiwan; 5 Department of Genomic Medicine, National Cheng Kung University Hospital, College of Medicine National Cheng Kung University, Tainan, Taiwan

**Keywords:** Metformin, Multiple acyl-coA dehydrogenase deficiency, Thyroid hormones, Topiramate

## Abstract

**Background:**

Multiple acyl-CoA dehydrogenase deficiency (MADD) is a treatable lipid metabolism disorder that presents as myopathy and episodic metabolic crisis. The metabolic crisis is typically associated with prolonged fasting or physical stress; however, the mechanism of metabolic crisis is not yet fully understood.

**Case presentation:**

A 28-year-old Taiwanese woman presented with dyspnoea, poor appetite, and muscle weakness after using antiobesity drugs, including metformin, triiodothyronine, and topiramate. MADD was diagnosed, and her symptoms rapidly improved after treatment with riboflavin, carnitine, and ubiquinone. To date, antiobesity drugs have not been reported to be a provoking factor in fatty acid oxidation disorder.

**Conclusions:**

The increase of β-oxidation activity due to antiobesity drugs supports the hypothetical substrate competition model for MADD metabolic crisis. Because the drugs our patient used are commonly prescribed, we report this case to increase the vigilance and proactivity of clinicians in recognising this treatable adult-onset myopathy.

## Background

Multiple acyl-CoA dehydrogenase deficiency (MADD) is a lipid metabolism disorder caused by defects in electron transfer flavoprotein or electron transfer protein dehydrogenase, encoded by *ETFA, ETFB*, and *ETFDH*. MADD is the most prevalent lipid storage disease in Taiwan, where the hotspot mutation *ETFDH* c.250G > A has a carrier frequency of 0.8% [1]. Patients with late-onset MADD typically present with lipid storage myopathy and episodic metabolic crisis. Metabolic crisis is usually associated with prolonged fasting or physical stress and manifests as the exacerbation of weakness, lethargy, vomiting, hypoketotic hypoglycaemia, and metabolic acidosis. The mechanism of metabolic crisis is not yet fully understood. Herein, we report the case of a patient with late-onset MADD who had an episodic exacerbation of muscle weakness after taking antiobesity drugs. This provoking factor has yet to be addressed in the literature. All possible mechanisms were reviewed and discussed. This report was approved by the Institutional Review Board of National Cheng Kung University Hospital (IRB Approval No. A-EC-108-009).

## Case presentation

The 28-year-old Taiwanese woman whose condition is reported herein had an uneventful birth and developmental history. She exhibited suboptimal performance in exercise since elementary school age, especially in long-distance running. She experienced one episode of reversible proximal limb weakness for 3 days when she was 21 years old. She denied any weakness at baseline. She used antiobesity drugs, and in the preceding 4 months, her body weight decreased from 60 to 47 kg, with a corresponding reduction in body mass index from 22.3 to 17.5. The regimen of antiobesity drugs included metformin, triiodothyronine, topiramate, pseudoephedrine, hydrochlorothiazide, fluoxetine, and oxazolam. She was not prescribed diet change or caloric restriction. After weight loss, she exhibited poor appetite; poor oral intake; dyspnoea on exertion; and progressive weakness and soreness in the four proximal limbs, the neck, and the trunk. She was aided by a wheelchair upon admission.

**Fig. 1:**
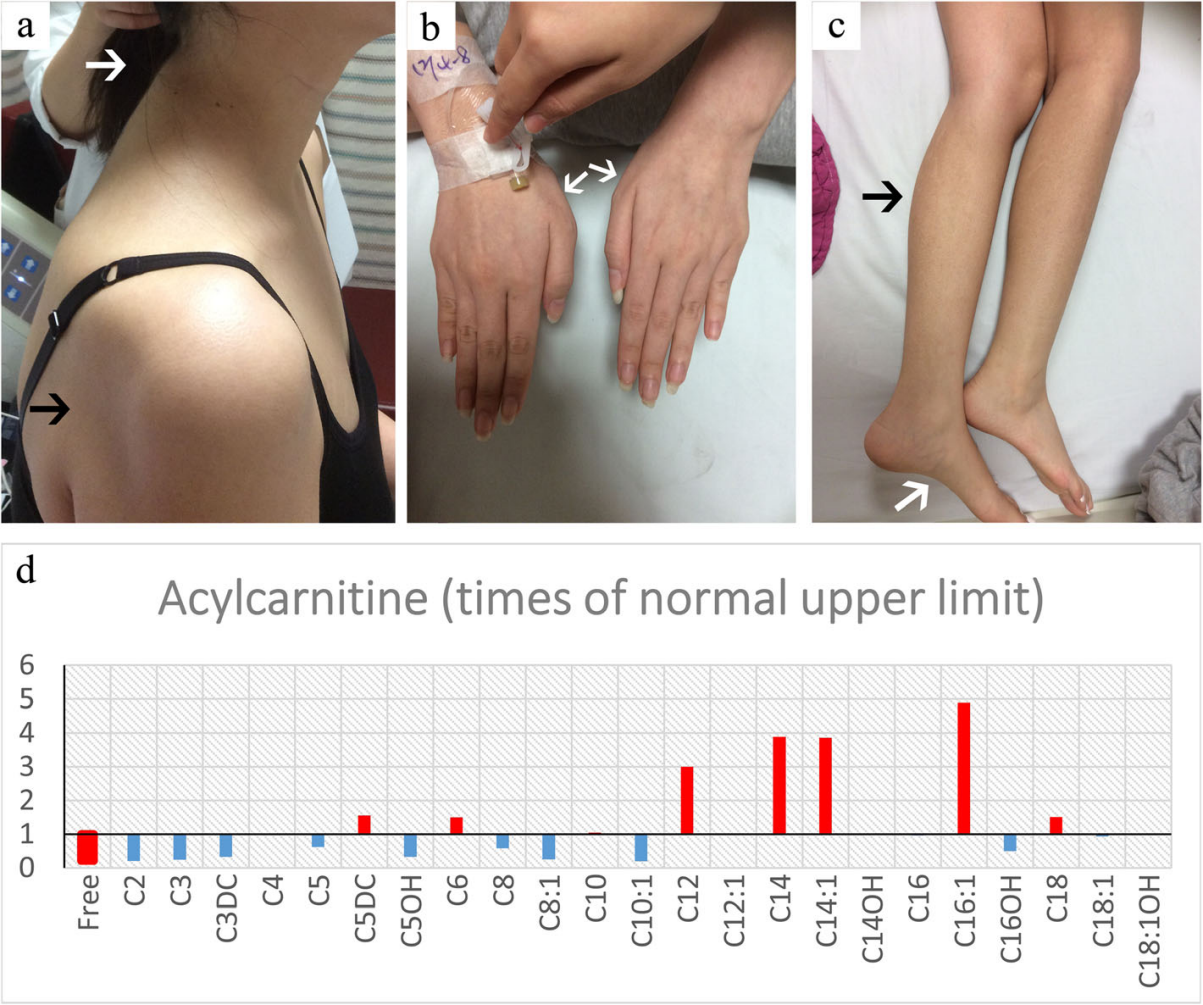
Clinical information. The patient exhibited prominent atrophy in the muscles of the neck (**a**, white arrow), shoulder girdle (**a**, black arrow), bilateral first dorsal interosseous muscles (**b**, white arrows), bilateral legs (**c**, black arrow), and feet (**c**, white arrow). Tandem mass spectrometry of serum acylcarnitine revealed elevated short-, medium-, and long-chain acylcarnitines with secondary carnitine deficiency (**d**, X axis, serum acylcarnitine with various chain lengths. Y axis, ratio of the serum acylcarnitine level of this patient and the upper limit of the normal range in those of equivalent age)

On examination, the patient had intermittent tachycardia and dyspnoea on minimal exertion but had otherwise normal vital signs. She had reduced muscle strength in the proximal limbs (manual muscle power: bilateral deltoid: 3; bilateral finger abduction: 5; bilateral hip flexion: 4; bilateral dorsiflexion: 5), neck, and trunk, with positive Gowers’ sign and myopathic gait. Generalised muscle wasting involving the neck, trunk, shoulder girdle, pelvic girdle, and proximal and distal limbs but not the facial muscles was observed (Fig. 1a-c). No percussion myotonia was noted. The patient had mildly decreased memory and calculation abilities compared with healthy individuals of an equivalent age. Examinations for cranial nerve, deep tendon reflex, sensory, and coordination function revealed unremarkable results. She had no hepatosplenomegaly, goitre, or skin hyperpigmentation.

**Fig. 2:**
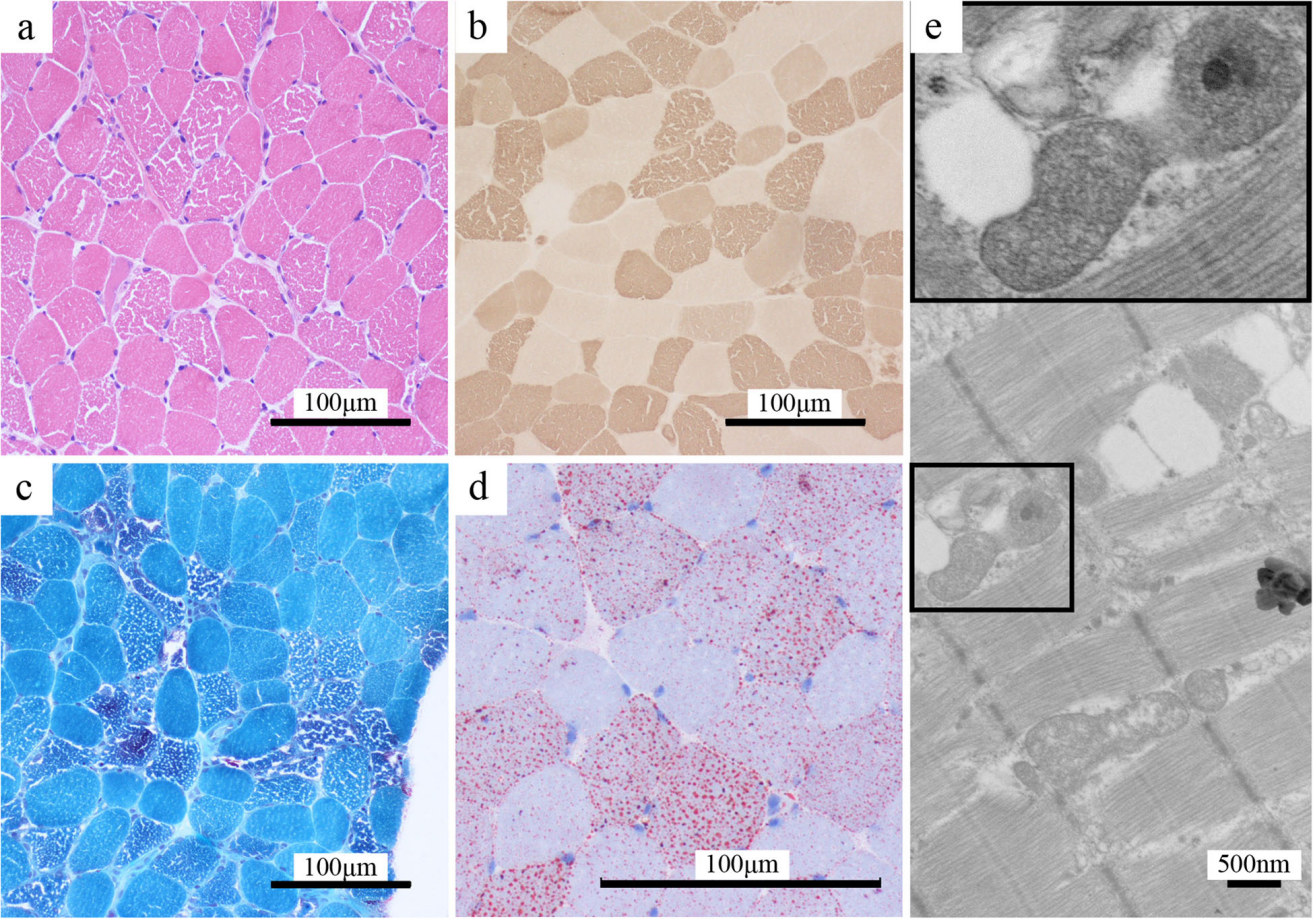
Muscle pathology. The haematoxylin and eosin slide indicated mildly varied muscle fibre size and scattered small vacuoles (**a**). The ATPase stain (pH 4.6) demonstrated that the vacuoles were mainly in type 1 fibres (**b**, dark colour fibres). The modified Gomori trichrome stain showed no ragged red fibres (**c**). The oil red O stain revealed increased lipid droplets in size and number in scattered fibres (**d**, tiny red droplets). Transmission electron microscopy indicated increased lipid droplet and mitochondria counts (**e**), with abnormal cristae and some dense inclusion in mitochondria (**e** inset). (Scale bar, 100 μm in B1–B4 and 500 nm in B5)

Laboratory exams indicated elevated serum creatinine kinase (496 U/L), elevated aspartate aminotransferase (102 U/L), elevated resting serum lactate (4.7 mmol/L), and borderline elevated serum calcium (10.2 mg/dL) levels. Blood count, estimated glomerular filtration rate, serum electrolyte levels (including sodium, potassium, and magnesium), and thyroid function were unremarkable. An arterial blood gas test revealed no hypoxemia, hypercapnia, or acid–base problems. A nerve conduction study yielded no evidence of polyneuropathy. Electromyography demonstrated myopathic motor unit action potential with early recruitment pattern as well as no spontaneous activity in all sampled muscles in the four proximal and distal limbs. An aerobic forearm exercise test [2] revealed elevated mixed venous oxygen saturation after 3 min of aerobic exercise, suggesting defective oxidative metabolism in the muscles (Table 1). Muscle magnetic resonance imaging of the bilateral thighs was unremarkable. Muscle pathology of the right rectus femoris muscle suggested lipid storage myopathy (Fig. 2). Tandem mass spectrometry of serum acylcarnitine revealed elevated short-, medium-, and long-chain acylcarnitines with secondary carnitine deficiency (Fig. 1d). Urine organic acid analysis indicated glutaric aciduria. *ETFDH* gene analysis revealed compound heterozygous mutations c.250G > A / p.Ala84Thr and c.524G > T / p.Arg175Leu. Both variants have previously been reported to be pathogenic [1, 3]. MADD was therefore diagnosed. We treated the patient with riboflavin, carnitine, and ubiquinone, and her symptoms rapidly improved. She was able to walk without assistance within 2 weeks, and she became asymptomatic within 3 months.

**Table 1:** Aerobic forearm exercise test. PvO2, lactate, pyruvate, and lactate/pyruvate ratios before and after exercise

	PvO2^a^ (mmHg)	Lactate (mmol/L)	Pyruvate (μmol/L)	Lactate/pyruvate
Pre-exercise	46	2.1	116	18.1
Post-exercise 0 min	62	5.1	118	43.2
Post-exercise 8 min	52	2.6	132	19.7

^a^PvO2 mixed venous oxygen saturation

## Discussion and conclusions

MADD is a lipid metabolism disorder that is caused by defects in electron transfer flavoprotein, which is encoded by *ETFA* and *ETFB*, or defects in electron transfer flavoprotein dehydrogenase, encoded by the *ETFDH*. Electron transfer flavoprotein and electron transfer flavoprotein dehydrogenase are located on the matrix face of the inner mitochondrial membrane and are involved in the oxidation of fatty acids. The aforementioned defects disturb electron transfer from acyl-CoA dehydrogenase (ACAD) through flavoproteins to ubiquinone and finally result in an accumulation of acyl-CoA of various chain lengths.

Late-onset MADD typically presents as lipid storage myopathy and episodic metabolic crisis. The myopathy usually manifests as proximal muscle weakness. Cardiomyopathy and respiratory insufficiency have also been reported. Metabolic crisis typically manifests as the exacerbation of weakness, lethargy, vomiting, hypoketotic hypoglycaemia, and metabolic acidosis. Common precipitating factors of acute exacerbation include prolonged fasting or physical stress such as exercise or infection [4]. To date, antiobesity drugs have not been reported as a provoking factor. Regarding the possible mechanisms by which the drugs induced metabolic crisis, this report supports the hypothetical model of substrate competition in disorders involving β-oxidation dysfunction. In the substrate competition model of fatty acid oxidation, the accumulation of acyl-CoA of a certain chain length acts as competitive inhibitor of ACADs of different chain lengths [5]. Therefore, the accumulation of acyl-CoA with multiple chain lengths exacerbates the dysfunction of multiple ACADs and forms the basis of metabolic crisis [6]. Moreover, coenzyme A may be trapped as acyl-CoA and lead to dysfunction of glucose metabolism, which results in profound energy failure [7]. Chokchaiwong et al. studied the mitochondrial bioenergetics of cells with *ETFDH* mutation and observed reduced mitochondrial membrane potential upon fatty acid challenge, supporting the concept of substrate competition in vitro; these researchers also reported decreased mRNA and protein levels of *ETFDH* upon fatty acid challenge, suggesting a complex negative feedback loop governing the pathophysiology of metabolic crisis [8].

Antiobesity drugs accelerate lipid catabolism. Microcosmically, they act comparably to a fatty acid challenge towards mitochondria, as in the aforementioned in vitro cell model. Metformin, one of our patient’s antiobesity drugs, is an electron transport chain complex I inhibitor, which activates AMP-activated protein kinase (AMPK) by increasing the AMP:ATP ratio. The AMPK pathway accelerates fatty acid transportation into mitochondria, which is typically the rate-limiting step of fatty acid oxidation [9]. Thyroid hormone directly enhances lipid oxidation by increasing AMPK pathway activity, carnitine palmitoyl transferase activity, flavin adenine dinucleotide-linked respiratory pathway activity, and mitochondrial trifunctional protein activity [10]. Topiramate increases fatty acid oxidation by increasing lipoprotein lipase activity and adiponectin levels and thus AMPK pathway activity [11]. Taken together, the results indicate that the acute exacerbation of muscle strength after antiobesity drug use likely resulted from an overload of fatty acid oxidation at multiple steps in a suboptimal lipid metabolism system. This supports the notion of substrate competition.

This case report has two main strengths. First, this is the first reported case of a patient with MADD who had in vivo lipid challenge induced by medication, which supports the substrate competition model. Second, the diagnosis in this case was comprehensively confirmed through genetic and metabolic diagnosis, and complete improvement after treatment was verified. Nevertheless, this report has two limitations. First, the patient used multiple antiobesity drugs simultaneously; thus, inferring which drug contributed most to the exacerbation is challenging. Second, reproducing the exacerbation episode was infeasible for ethical reasons.

The medications this patient used, including metformin, thyroid hormone, and topiramate, are commonly used in clinical practice and can affect lipid metabolism, resulting in acute decompensation in patients with MADD. Thus, we report this case to increase the vigilance and proactivity of clinicians in recognising this treatable form of adult-onset myopathy.

## Data Availability

Data sharing is not applicable to this article because no datasets were generated or analysed in the current study.

## Abbreviations

MADD: Multiple acyl-CoA dehydrogenase deficiency
ACAD: Acyl-CoA dehydrogenase
AMPK: AMP-activated protein kinase
